# The Good Old Times

**Published:** 1910-07-30

**Authors:** 


### SPECIAL ARTICLES.

### "THE GOOD OLD TIMES."

Some idea of the horrors and barbarities of medical treatment a couple of centuries ago may be gathered from an interesting article by Dr. van Leersum which appears in the June number of *Janus*, a periodical devoted to medical history and geography. The author devotes his paper to a couple of letters of Gerard van Swieten on the "Liquor Swietenii" and to the history of the "Inoculation of small-pox in Holland and the Low Countries."

It was towards the end of the fifteenth century that the use of mercury and its compounds in the treatment of syphilis first came into vogue, but the physicians only introduced the drug into their practice after its use had been allowed to run riot at the hands of quacks and of those who were then classified in the same category—the surgeons. It was given externally either as a vapour or in an ointment. Much opposition was arrayed against the treatment, many believing that fistulæ and swellings were produced by the drug. Ignorance of its poisonous qualities and the humoral pathological explanation of its action certainly made mercury a drug to be feared, and since "salivation" was taken as the criterion of its efficacy, with consequent "pushing" of the treatment to its utmost limits, the condition of the unfortunate patient can be better imagined than described. "Foetor ex ore" was the token of salutary action. Even the comparatively mild treatment of Paré, who recognised the injurious effects of mercury, would be heroic to-day. Boerhaave, who never pushed salivation to excess, thought with others that the easiest channel for the elimination of the syphilitic virus was the salivary glands. Treatment "secundum artem" was at that time both costly and dangerous. A rigid preliminary dieting, followed by a course of severe purging, led up to bleeding and sweating "cures." The patient, being then in a condition to receive the drug, was confined continuously to a room practically hermetically sealed for 20 to 30 days, during which he was not allowed to remove his clothes!

Dr. van Leersum, on the authority of Delaunay, mentions the case of Sieur Van den Mersche and his lady, who, coming up to Paris from Flanders in 1731 for anti-syphilitic treatment, consulted no fewer than six physicians. "Le Grand Remède" was decided upon, and the good-natured gentleman determined to undergo the ordeal first in order to encourage his wife. Prepared for all eventualities, after settling his affairs, he received the Sacrament, and treatment was commenced by Cernaizot, one of the six consultants. His formula consisted of "Clysterium donare, postea seignare, ensuita purgare," which he followed up by "reseignare, repurgare, et reclysterisare." Having accomplished all these things to his satisfaction, he rubbed the patient with "six gros d'onguent mercuriel," alternating on the following days with two and with one "gros" of ointment. Eight days later the patient had a fit, during which the hastily summoned Seron (another of the six) bled him several times in succession, but without avail. To save the good name of the deceased gentleman, Seron gave out that death was due to apoplexy, while to save the widow from despair he forthwith proceeded to marry her. But this was not the end of the tragi-comedy, for the relative of the deceased, fearing that a stranger might appropriate the inheritance, instituted proceedings in the law courts to secure the property! History, unfortunately, is silent as to the result of their pleadings. It must not, however, be imagined that such barbarity was peculiar to the French school of practitioners, for the Viennese faculty acted much in the same manner, and it was only after a prolonged struggle on the part of Van Swieten that some degree of humanity commenced to tinge the therapeutic methods of the time. By reason of his popularity with Maria Teresa and his important position as Censor, his opinions carried weight in excess of that of his contemporaries. While not the first to recognise that salivation was unnecessary to the production of a cure, he pointed out that external administration gave no criterion of the amount of mercury absorbed, an idea which again was not original. After trying various mercurial compounds, he hit upon "sublimated mercury," a drug which had been previously administered for syphilis by an old surgeon whose name is forgotten. The prescription which bears Van Swieten's name, which is still to be found in the pharmacopœias of several countries, and for which, be it said, he claims no originality, is practically identical with that of the old surgeon. It was but natural that a person in his exalted position should have many enemies, and at first his formula was decried, as much, perhaps, on account of faulty administration as from malice. Sublimate is as poisonous as calomel and mercury, and all three produce salivation. In the end, however, his method prevailed and was gradually adopted throughout Europe. It is only of late that the "liquor Swietenii" has fallen into disuse, but its active principle, "sublimate," still continues to hold its own among a host of preparations recommended in the treatment of syphilis.

Both the letters which Dr. van Leersum prints are from Van Swieten to Dr. van Leempoel, of Rotterdam, and both are with reference to the remedy. The first is dated July 23, 1755; the second, April 18, 1757. A translation in English is given of both, as well as the original Dutch. A facsimile copy of the first is also printed, from which it can be seen that the writer possessed a clear, well-formed handwriting with plenty of character. The interesting paper is brought to a close by an account of the spread in Holland of the inoculation treatment of small-pox—a spread in which Van Swieten's correspondent, Van Leempoel, took an active part.

